# Why Turner patients with 45, X monosomy should not be excluded from fertility preservation services

**DOI:** 10.1186/s12958-022-01015-z

**Published:** 2022-09-22

**Authors:** MJ Schleedoorn, K Fleischer, DDM Braat, AJM Oerlemans, AAEM van der Velden, R Peek

**Affiliations:** 1 Obstetrics and Gynaecology, Radboud University Medical Centre, Nijmegen, the Netherlands; 2 Medical Ethics, Radboud University Medical Centre, Nijmegen, the Netherlands; 3 Paediatric Endocrinology, Radboud University Medical Centre Amalia Children’s Hospital, Nijmegen, the Netherlands

**Keywords:** Turner syndrome, Fertility presentation, Fertility counselling, Ovarian tissue cryopreservation, Daily practice challenges

## Abstract

In this case report, we highlight the practical dilemma, i.e. to perform ovarian tissue cryopreservation surgery in a 45, X Turner Syndrome patient or not, by reporting on the presence of follicles in a 13-year-old female diagnosed with 45, X monosomy and an unmeasurable anti-müllerian hormone serum level. We compare our results with previous research, highlight the challenges we faced in this case and provide recommendations for daily practice. Hereby, we demonstrate that excluding certain subgroups of Turner Syndrome patients (e.g. monosomy patients, and/or girls with an anti-müllerian hormone level below 2.0 ng/l) may be premature, especially based on the current state of published research data. This practical example of a challenging dilemma in the counselling of Turner Syndrome patients for fertility preservation is of interest for clinicians involved in fertility counselling and Turner Syndrome care.

## Introduction

An estimated 1 in 2,500 girls are born with Turner syndrome (TS). TS involves the partial or complete absence of a second sex chromosome. The most common variant is 45, X, in which a second sex chromosome is missing. When this applies to all analysed somatic cells, the condition is called 45, X monosomy. However, some women are missing a second X chromosome in only some of their cells, and yet have a ‘normal’ 46, XX pattern or a 47, XXX pattern in other cells. This is referred to as 45, X / 46XX or 45, X / 47, XXX / 46, XX mosaicism. Finally, in some patients, TS is caused by a structural abnormality of one of the two X chromosomes.

One of the most common features of TS is early menopause which is caused by an accelerated degeneration of the oocytes in the ovaries. In most women with TS this results in a premature exhaustion of the ovarian reserve at the time they wish to become a parent. The inability to bear a biological child is one of the most painful challenges endured by females with TS, regardless of their age [1, 2]. Even though the majority of adult females with TS embrace alternative options to become a parent such as adoption [1], healthcare professionals are increasingly asked for the possibility to overcome infertility by experimental fertility preservation techniques (practical experience).

Fertility preservation includes the cryopreservation of the patient’s own gametes, either by preserving mature oocytes or ovarian tissue containing primordial follicles. Cryopreservation of mature oocytes (OC) is an established fertility preservation approach but can be performed only in post-pubertal females, as it requires ovarian activity and psychological maturity. This means that OC would be limited to only a small percentage of females with TS, i.e. those girls with a spontaneous onset puberty and regular menstruation who are emotionally mature enough to undergo the procedure.

The second technique, ovarian tissue cryopreservation (OTC), has the disadvantage that it requires surgery, most preferably a laparoscopic ovarian biopsy, ovarian wedge resection, or a unilateral ovariectomy. However, OTC can be performed regardless of the patient’s age or pubertal development, and thus appears to be a promising technique to preserve the fertility of young girls with TS [3]. It provides the option to store primordial follicles within the ovarian tissue before their premature disappearance.

The efficacy of OTC in other patient groups has been well-described, and over the past decades, several guidelines have been developed. Retransplantation of cryopreserved-thawed ovarian cortical tissue in non-TS patients has resulted in restoration of ovarian function [4, 5], pregnancies, and healthy offspring [6–11].

Whether OTC in females with TS will lead to healthy offspring is currently unknown. In the past 20 years, freezing ovarian tissue has been applied to more than 100 girls with TS. These studies only describe the presence or absence of oocytes. There have been no reports thus far describing the autotransplantation of previously frozen ovarian tissue in girls with Turner, and let alone whether this has resulted in pregnancies and live births.

Based on earlier studies on the presence or absence of oocytes in ovarian tissue, clinicians suggest that certain subgroups with TS should be excluded from experimental fertility preservation services [3, 12, 13]. The most frequently mentioned exclusion groups are the 45, X monosomy patients, and girls with an [14] anti-müllerian hormone (AMH) serum level below 2.0 ug/l.

We believe that excluding certain subgroups solely based on either their genotype or one suboptimal hormone parameter might be premature based on the current state of research data. To demonstrate this, we describe a practical example of a challenging dilemma in the counselling of TS girls for fertility preservation by using a case from our daily practice.

## Case report

We report on a 13-year-old female who had recently been referred to our centre for specialized fertility counselling. At the age of 8 years she was diagnosed with TS upon a diagnostic work-up for growth retardation. Chromosome analysis on 30 peripheral blood lymphocytes revealed a 45, X monosomy, or classical TS karyotype. No other TS-associated problems were detected and growth hormone therapy was started. Surprisingly, she presented with the first signs of spontaneous pubertal development, i.e. breast gland development (thelarche), at the age of 10 years. Two years later she reported monthly periods of 5–7 days (non-cyclical) vaginal bleeding. Endocrinological investigation showed normal Follicle Stimulating Hormone (FSH) levels (6.1–9.5 E/l; ref 1.7–21.5 E/l), normal Oestradiol (E2) levels (74–100 pmol/l; ref 45–1461 pmol/l), normal Luteinizing Hormone (LH) levels (1.2–2.0 E/l; ref 1.0–95.6 E/l), and a low AMH level (0.2 ug/l; ref 0.56–8.4 µg/l) (Table 1).

**Table 1:** Overview of endocrinological assessments

**Time of endocrinological assessment**	*Referral*	*Counselling*	*Surgery*	*- yea- post-surgery*	*- year post-surgery*
Patient’s age	12-years-old	13-years-old	13-years-old	14-years-old	15-years-old
Anti-müllerian hormone (**AMH**) level; *ref 0.56–8.4 µg/l*	0.2	< 0.10	0.1	< 0.10	< 0.10
Follicle Stimulating Hormone (**FSH**) level; *ref 1.7–21.5 E/l*	6.1–9.5	8.1	23	8.8	84
Luteinizing Hormone (**LH**) level; *ref 1.0–95.6 E/l*	1.2–2.0	n/a	7.3	2.3	40
Oestradiol (**E2**) level; *ref 45–1.461 pmol/l*	74–100	n/a	< 18	89	< 18

At the time she was referred for fertility counselling, her AMH level was < 0.1 ug/l (ref 0.56–8.4 µg/l) (Table 1) and she had been having irregular periods of vaginal bleedings since a few months. Her FSH level remained normal (8.1 E/l; ref 1.7–21.5 E/l). No other endocrinological parameters were determined. Her last physical examination revealed a Tanner stage P2, M3. Transabdominal sonography was performed in order to visualize the genitalia interna. The uterus appeared to be anteverted, with a normal size for the patient’s age and an endometrial thickness of 11 mm. The antral follicle count (AFC) in the left ovary was 4, and no cysts or free fluid was reported. The right ovary could not be visualized.

In order to exclude the presence of a Y-cell line but also to properly inform the girl about her fertility chances, a FISH analysis was performed on buccal cells following the standard protocols [15]. Surprisingly, a 47, XXX [78] / 45, X [33] / 46, XX [7] karyotype was reported for these cells. This additional FISH examination showed that this was not a full-blown monosomy case but a patient with hidden mosaicism.

The history of the patient combined with the additional collected information pointed most in the direction of a very low ovarian reserve. We discussed OTC in an experimental setting (Trial registration number NCT03381300) but also mentioned alternative options such as wait and see, and future options such as adoption, foster parenting or trying to conceive with an oocyte donor. Oocyte vitrification was also discussed, but not preferred because of the patient’s age and her diminished ovarian reserve.

After comprehensive counselling, the girl and her parents decided for experimental OTC. Although transabdominal sonography is not conclusive for the presence of ovaries, we discussed the plausible scenarios of an absent or streak right ovary. Informed consent was obtained to remove the ‘normal-looking’ ovary in the case that the ovarian left–right difference would be confirmed during the laparoscopic intervention.

### Ovarian tissue cryopreservation

**Fig. 1:**
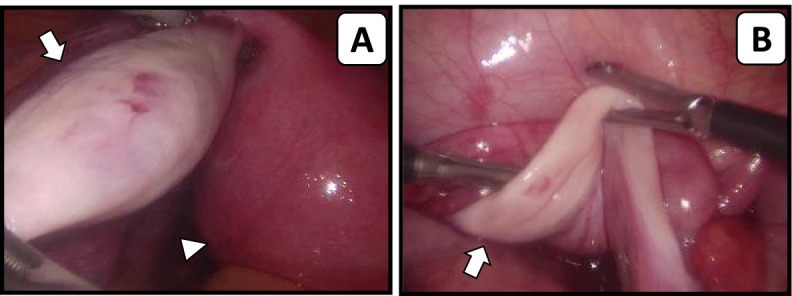
Laparoscopic images of the genitalia interna. **A** Normal left ovary (arrow) and uterus (arrow head) (**B**) streak right ovary (arrow)

During laparoscopy, four months later, a normal sized uterus was seen and the difference in ovarian volume was confirmed with a macroscopically ‘normal-looking’ left ovary and a streak right ovary (Fig. 1). The left ovary could be removed for fertility preservation purposes without any complications.

Serum hormone concentrations were measured just before the ovariectomy was performed and showed an elevated FSH serum concentration of 23 E/l (ref 1.7–21.5 E/l), an E2 concentration under the laboratory detection level (i.e. < 18 pmol/l), while the AMH level remained under the reference value (0.1 ug/l; ref 0.56–8.4 µg/l) (Table 1).

Ovarian tissue could be cryopreserved successfully according to the local protocol [16]. One representative fragment was kept aside and used for histological and FISH analysis of ovarian cells as described previously [17].

**Fig. 2:**
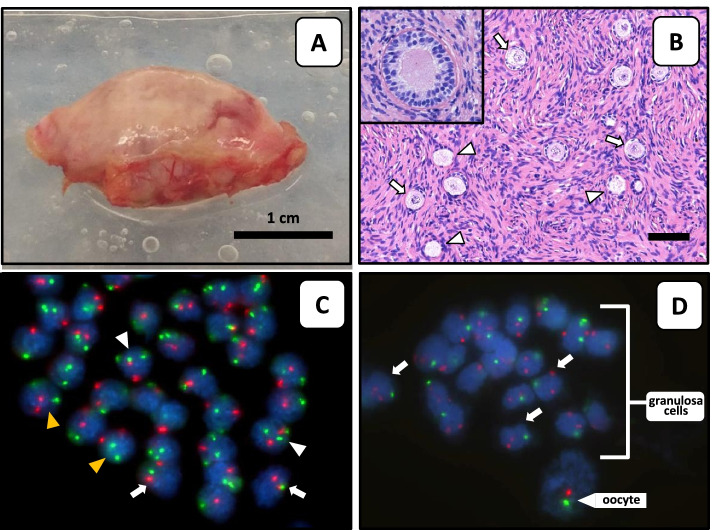
Macroscopic, histologic and FISH analysis of ovarian tissue. **A** Directly after unilateral ovariectomy the ovary was transported to the laboratory for preparation of cortex fragments for fertility preservation purposes. **B** Haematoxylin–eosin stained section of cortex tissue with empty follicles (arrows) and follicles with missing granulosa cells (arrow heads). The inset shows a multilaminar follicle with no recognizable oocyte. FISH analysis of cortex stromal cells (**C**) and of cells from a single follicle (**D**) with X-chromosome (green) and chromosome 18 (red) specific probes. Arrows point to 45, X cells, white arrow heads to 47, XXX cells and yellow arrow heads to 46, XX cells. The FISH signals from the oocyte are indicated (**D**). Note that FISH signals are not all in the same focus plane

Histological analysis showed a decreased follicular density (7.0 follicles/mg tissue) as compared with the ovarian tissue of same-aged non-TS females undergoing fertility preservation [18]. Remarkably, several empty primordial follicles were seen, and even a multilaminar follicle without a recognizable oocyte (Fig. 2). Furthermore, the tissue contained multiple primordial follicles with an incomplete layer of granulosa cells (Fig. 2).

**Fig. 3:**
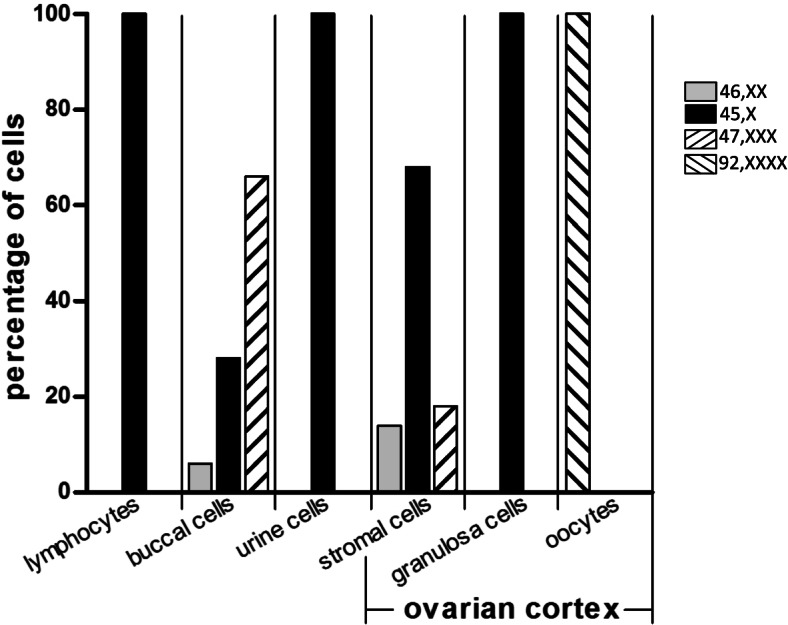
Mosaicism in extra-ovarian tissues and ovarian stromal cells, granulosa cells and oocytes. Lymphocytes (*n* = 30), cells from buccal smears (*n* = 100) and urine (*n* = 100) were analysed by FISH with an X chromosomal peri-centromeric probe. The percentage of cells with a particular karyotype is indicated. In addition, the X-chromosomal content of ovarian cortex components including stromal cells (*n* = 80), granulosa cells (*n* = 262 from 8 individual unilaminar follicles) and oocytes (*n* = 6) is shown. Three different karyotypes (45, X / 46, XX / 47, XXX) were detected in the somatic cells while all oocytes were tetraploid (92, XXXX)

FISH analysis of the stromal cells (*n* = 80) showed a mosaic pattern with 3 cell lines 45, X [68] / 47, XXX [18] / 46, XX [14], while a full-blown 45, X karyotype was seen in the granulosa cells (*n* = 262) (Figs. 2 and 3). Remarkably, all 6 oocytes that were analysed showed a normal (tetraploid 92, XXXX) karyotype (Figs. 2 and 3).

In order to check if the X chromosome content of other cell types than lymphocytes or buccal cells was more representative for the karyotype of the ovarian cells, we also performed a FISH analysis on 100 urine cells according to the local protocol [17]. However, all cells that were investigated (*n* = 100) showed a 45, X karyotype (Fig. 3), and were thus, representative for the karyotype of the granulosa cells, but not for the oocytes or stromal cells in this patient. Recently, similar findings in other females with TS have been reported [17, 19]. The functional consequences of these aneuploid granulosa cells for the follicular development is currently unknown.

### Follow-up

Surprisingly, the patient reported monthly periods of vaginal bleeding for the first year after the operation. Furthermore, endocrinological blood examination now showed normal gonadotropic hormone levels (FSH 8.8 E/l; ref 1.7–21.5 E/ and, LH 2.3 E/l; ref 1.0–95.6 E/l) although her E2 was relatively low (89 pmol/l, ref 45–1461 pmol/l.). Her AMH level remained under the reference value (Table 1). No other specific details were reported. Two years after surgery, physical examination showed complete pubertal development. However, she was now again reporting episodes of irregular blood loss. Transabdominal sonography showed a normal uterine size for the patient’s age and an endometrial thickness of 4 mm. The right ovary could not be visualized. Unfortunately, the serum hormone concentrations revealed hypergonadotropic hypogonadism with elevated FSH (84 E/l, ref 1.7–21.5 E/l) and LH (40 E/l, ref 1.0–95.6 E/l) levels, and an unmeasurable E2 (< 18 pmol/l) and AMH concentrations (< 0.10) (Table 1). No climacteric symptoms were reported. Nevertheless, the repeatedly measured low oestrogen value based on a confirmed hypergonadotropic hypogonadism status was an indication to start hormone replacement therapy 3 years after surgery. We agreed with the patient to return at least once a year to our combined gynaecology-and-endocrinology clinic for further guidance and follow-up.

## Discussion

In this case report, we highlight the practical dilemma (i.e. to perform OTC surgery in a 45, X TS patient or not) by reporting on the presence of follicles in a 13-year-old female diagnosed with 45, X monosomy and an unmeasurable AMH. We compare our results with previous research, highlight the challenges we faced in this case and provide recommendations for daily practice. Hereby, we demonstrate that excluding certain subgroups of TS patients (e.g. monosomy patients, and/or girls with an AMH below 2.0 ug/l) may be premature, especially based on the current state of published research data.

From previous research [3, 20, 21], we know that the presence of a 46, XX cell line and a measurable AMH level have been associated with spontaneous pubertal development, the presence of follicles and spontaneous pregnancies in TS patients. However, optimal discriminative markers for the presence or absence of follicles in females with TS are currently still lacking. AMH serum levels can vary considerably due to biological variability and intertest variability of AMH assays, and there are limited prospective longitudinal data assessing AMH concentrations in prepubertal girls. Therefore, adequate interpretation of AMH levels, especially in prepubertal TS patients, is difficult. Furthermore, most research articles on TS, including key papers that are describing the presence or absence of follicles in females with TS, still lack a description of the methods used for genotyping [3, 22–25]. This is, however, essential information especially when linked to fertility or fertility preservation. Missing X chromosomal content in ovarian cells is likely to result in germ cell apoptosis and impaired folliculogenesis during foetal life [26, 27]. One could assume that the described cases where follicles were found or pregnancies have occurred in females with 45, X monosomy were actually examples of undetected germ cell mosaicism [28, 29].

In routine care, karyotyping of lymphocytes is the standard technique used to determine TS. most girls have been diagnosed with TS based on the examination of only one cell type. In most centres, the diagnosis is based on 20–30 lymphocytes only, although it has been recommended to determine the karyotype of at least 50 metaphases to exclude < 10% of mosaicism (CI 0.99) [30]. Interestingly, a recent cohort study in 142 adult patients with TS, revealed that the percentage of 45, X cells in lymphocytes and buccal cells was identical in less than one third of cases [31]. For this reason, some experts [15, 31, 32] recommend adding buccal cell FISH analysis for a better diagnosis-prognosis-treatment and guidance for some subgroups of TS patients.

Our patient had been diagnosed with 45, X monosomy but presented with spontaneous pubertal development and monthly periods of vaginal bleeding (5–7 days). Additional FISH examination of buccal smear revealed mosaicism. At the time she was referred to us, she was reporting irregular periods of vaginal bleeding since a few months and the endocrinological assessment repeatedly showed an AMH level under the reference value. Since the ovarian function in TS patients only declines with age, it seemed likely that this girl had diminished ovarian reserve. The counselling of the girl and her parents therefore included a difficult dilemma: should we perform OTC surgery or not?

During the counselling process by a dedicated team, different considerations were discussed and addressed.

First of all, we must be careful not to raise false hopes even if OTC might be the only possibility to preserve the ovarian follicles that are still left. In this patient, it was questionable whether the ovarian reserve in the normal-looking ovary would still be sufficient for fertility preservation purposes. In other words: if there is already an imminent premature ovarian insufficiency (POI), does it make sense to later transfer the frozen and thawed pieces back? Especially since the freezing and thawing of ovarian tissue will also have a negative effect on the number of viable oocytes and thus the chance of pregnancy and live birth. In addition, patients with TS are known to have a higher risk of having a miscarriage [20]) and a slightly higher risk of having a child with a congenital disorder [20, 33]. Therefore, not only the quantity but also the functionality of the oocytes and supporting ovarian cells must be considered. In vitro* activation (IVA)* [34] or in vitro* maturation (IVM)*, eventually combined with preimplantation genetic diagnosis (PGD) [35] could possibly improve the chances of pregnancy and live birth in the future. However, both techniques (e.g. IVA and IVM) are at the moment still experimental. Furthermore, we should also be aware of the greater frequency of uterine morphological anomalies in patients with TS and the potential for compromised endometrial receptivity [20].

The second consideration in this case was whether we are not reducing her chances of a (complete) spontaneous pubertal development by performing an ovariectomy. Unfortunately, there are no follow-up studies that report on the effect of ovarian surgery on the chances of spontaneous pubertal development, pregnancy and risk of (earlier) menopause in patients with TS. In non-TS patients, an ovariectomy does not affect the chances of pregnancy [36] but may lead to 1–3 years earlier onset of menopause [37]. However, these data are obviously from girls and women with normal ovarian reserve, and the remaining ovary might ‘compensate’ for the removed ovary. The impact of this procedure on our patient is unknown, but most likely, her ovarian reserve would not be sufficient to become pregnant later, but possibly just enough to complete spontaneous pubertal development. We discussed that in the worst case, this process could be stagnated by the removal of the ‘normal’ looking ovary. This would mean that she, like many TS patients, would become dependent on hormone replacement therapy, but at an earlier time. Fortunately, this patient experienced complete pubertal development without hormone replacement therapy. In addition, she continued to have periods of (irregular) vaginal bleeding two years after surgery. However, these might have been anovulatory cycles with persistent follicles. Nevertheless, a repeatedly measured low oestrogen value based on a confirmed hypergonadotropic hypogonadism status was an indication to start hormone replacement therapy 3 years after surgery.

Lastly, the increased risk of foetal and maternal complications during future pregnancy and labour in females with TS should be considered. In comparison with healthy females, females with TS are at increased risk of intra-uterine growth restriction, preterm labour, thyroid dysfunction, diabetes, obesity, hypertension and pre-eclampsia [38]. In the past, women with TS were even discouraged to become pregnant due to the risk of aortic dissection. Recent studies have shown however, that this risk has decreased to 0.5% due to increased awareness of cardiovascular complications, improved preconception screening, and strict cardiovascular monitoring during pregnancy [39, 40]. Our patient had no history of aortic dissection, neither indications of an increased risk for aortic dissection, e.g. an increased aortic size index, bicuspid aortic valve, elongation of the transverse aorta, or coarctation of the aorta in combination with hypertension.

In conclusion, it seems that in this case, performing OTC was an appropriate decision. The ovariectomy did not affect her spontaneous pubertal development, and possibly we were just in time to preserve her fertility. Considering the low ovarian reserve, we found in the preserved ovarian fragments, it would have been very unlikely that she would have been able to conceive spontaneously in adulthood. Hereby, we demonstrate that excluding certain subgroups of TS patients from OTC (e.g. monosomy patients, and/or girls with an AMH below 2.0 ug/l) may be premature, especially based on the current state of published research data.

Our case is not the first case of a girl with TS who presented with a perioperative left–right difference in ovarian morphology during laparoscopic surgery [19]. However, this is the first case reporting on the presence of both a (morphological) streak ovary and normal ovary in a young female with TS. We recommend other clinicians to also discuss this rare outcome during fertility preservation counselling, and how to deal with it during laparoscopic surgery, even if we do not know yet what the clinical consequences are. In this patient, the left ovary could be visualized during transabdominal ultrasound (TUS). However, distinguishing precisely between anatomically normal ovaries and streak ovaries is not easy, and there might be a high chance that the ovaries cannot be visualized at all [41]. A Danish study showed that ovaries could be detected in 37% of young females with TS by TUS and in 55% by MRI (*P* = 0·1) [42], but they did not confirm their findings with laparoscopic surgery, which is considered to be the gold standard. We recommend further investigation of these imaging methods before applying these methods in daily practice.

As this is our 2nd case where we report on a girl who had been diagnosed with classical monosomy TS (45, X), but presented with a hidden X mosaicism in her ovarian cells [19], we want to highlight again the importance of buccal cell analysis for some subgroups of TS patients. In particular, in patients who have been diagnosed with 45, X monosomy to better differentiate between monosomy and mosaicism.

Moreover, we hope that future research reports will be more specific about the methods used for genotyping when reporting on TS and fertility or fertility preservation. We recommend including at least the type and number of cells on which the diagnosis is based. Lastly, by raising awareness about the possibilities of fertility preservation for girls with TS, we hope that more girls will be referred to a fertility specialist for fertility counselling. Since the current referral rates are rather low [14], we hope that by providing them with more information about their fertility and the full range of options available now and in the future to fulfil their desire to have children, will lead to better informed patients and less psychological burden.

## Data Availability

Additional files are available from the corresponding author on request.

## Abbreviations

AFC: Antral Follicle Count
AMH: Anti-Müllerian Hormone
CI: Confidence Interval
E2: Oestradiol
FFPE: Formalin Fixated and Paraffin Embedded
FISH: Fluorescence In Situ Hybridization
FSH: Follicle-Stimulating Hormone
HE: Haematoxylin and Eosin stain
IVA: In Vitro Activation
IVM: In Vitro Maturation
LH: Luteinizing Hormone
MRI: Magnetic Resonance Imaging
OC: Cryopreservation of mature Oocytes
OTC: Ovarian Tissue Cryopreservation
PGD: Preimplantation Genetic Diagnosis
POI: Premature Ovarian Insufficiency
TS: Turner Syndrome
TUS: Transabdominal Ultrasound
US: Ultrasound

## References

[1] Sutton EJ, McInerney-Leo A, Bondy CA, Gollust SE, King D, Biesecker B (2005). Turner syndrome: four challenges across the lifespan. Am J Med Genet A.

[2] Sylven L, Hagenfeldt K, Brondum-Nielsen K, von Schoultz B (1991). Middle-aged women with Turner's syndrome. Medical status, hormonal treatment and social life. Acta Endocrinol (Copenh).

[3] Borgstrom B, Hreinsson J, Rasmussen C, Sheikhi M, Fried G, Keros V (2009). Fertility preservation in girls with turner syndrome: prognostic signs of the presence of ovarian follicles. J Clin Endocrinol Metab.

[4] Oktay K, Karlikaya G (2000). Ovarian function after transplantation of frozen, banked autologous ovarian tissue. N Engl J Med.

[5] Radford JA, Lieberman BA, Brison DR, Smith AR, Critchlow JD, Russell SA (2001). Orthotopic reimplantation of cryopreserved ovarian cortical strips after high-dose chemotherapy for Hodgkin's lymphoma. Lancet.

[6] Donnez J, Dolmans MM, Demylle D, Jadoul P, Pirard C, Squifflet J (2004). Livebirth after orthotopic transplantation of cryopreserved ovarian tissue. Lancet.

[7] Andersen CY (2014). ESHRE campus "Fertility preservation: from technique to implementation in clinical practice".

[8] Demeestere I, Simon P, Dedeken L, Moffa F, Tsepelidis S, Brachet C (2015). Live birth after autograft of ovarian tissue cryopreserved during childhood. Hum Reprod.

[9] Dittrich R, Hackl J, Lotz L, Hoffmann I, Beckmann MW (2015). Pregnancies and live births after 20 transplantations of cryopreserved ovarian tissue in a single center. Fertil Steril.

[10] Meirow D, Levron J, Eldar-Geva T, Hardan I, Fridman E, Zalel Y (2005). Pregnancy after transplantation of cryopreserved ovarian tissue in a patient with ovarian failure after chemotherapy. N Engl J Med.

[11] Donnez J, Dolmans MM, Pellicer A, Diaz-Garcia C, Sanchez Serrano M, Schmidt KT (2013). Restoration of ovarian activity and pregnancy after transplantation of cryopreserved ovarian tissue: a review of 60 cases of reimplantation. Fertil Steril.

[12] Oktay K, Bedoschi G, Berkowitz K, Bronson R, Kashani B, McGovern P, et al. Fertility preservation in females with turner syndrome: a comprehensive review and practical guidelines. J Pediatr Adolesc Gynecol. 2016;29(5): 409–16. 10.1016/j.jpag.2015.10.011 PMC5015771 26485320

[13] Grynberg M, Bidet M, Benard J, Poulain M, Sonigo C, Cedrin-Durnerin I (2016). Fertility preservation in Turner syndrome. Fertil Steril.

[14] Morgan TL, Kapa HM, Crerand CE, Kremen J, Tishelman A, Davis S (2019). Fertility counseling and preservation discussions for females with Turner syndrome in pediatric centers: practice patterns and predictors. Fertil Steril.

[15] Freriks K, Timmers HJ, Netea-Maier RT, Beerendonk CC, Otten BJ, van Alfen-van der Velden JA (2013). Buccal cell FISH and blood PCR-Y detect high rates of X chromosomal mosaicism and Y chromosomal derivatives in patients with Turner syndrome. Eur J Med Genet.

[16] Peek R, Bastings L, Westphal JR, Massuger LF, Braat DD, Beerendonk CC (2015). A preliminary study on a new model system to evaluate tumour-detection and tumour-purging protocols in ovarian cortex tissue intended for fertility preservation. Hum Reprod.

[17] Peek R, Schleedoorn M, Smeets D, van de Zande G, Groenman F, Braat D (2019). Ovarian follicles of young patients with Turner's syndrome contain normal oocytes but monosomic 45. X granulosa cells. Hum Reprod.

[18] Liebenthron J, Reinsberg J, van der Ven K, Saenger N, Kruessel J-S, von Wolff M (2019). Serum anti-Müllerian hormone concentration and follicle density throughout reproductive life and in different diseases—implications in fertility preservation. Hum Reprod.

[19] Nadesapillai S, van der Velden J, Smeets D, van de Zande G, Braat D, Fleischer K, et al. Why are some patients with 45,X Turner syndrome fertile? A young girl with classical 45,X Turner syndrome and a cryptic mosaicism in the ovary. Fertil Steril. 2021;115(5):1280–87. 10.1016/j.fertnstert.2020.11.006 33342535

[20] Bernard V, Donadille B, Zenaty D, Courtillot C, Salenave S, Brac dela Perrière A (2016). Spontaneous fertility and pregnancy outcomes amongst 480 women with Turner syndrome. Human Reproduction.

[21] Lunding SA, Aksglaede L, Anderson RA, Main KM, Juul A, Hagen CP (2015). AMH as predictor of premature ovarian insufficiency: a longitudinal study of 120 Turner syndrome patients. J Clin Endocrinol Metab.

[22] Mamsen LS, Charkiewicz K, Anderson RA, Telfer EE, McLaughlin M, Kelsey TW (2019). Characterization of follicles in girls and young women with Turner syndrome who underwent ovarian tissue cryopreservation. Fertil Steril.

[23] von Wolff M, Dittrich R, Liebenthron J, Nawroth F, Schüring AN, Bruckner T (2015). Fertility-preservation counselling and treatment for medical reasons: data from a multinational network of over 5000 women. Reprod Biomed Online.

[24] Jadoul P, Guilmain A, Squifflet J, Luyckx M, Votino R, Wyns C (2017). Efficacy of ovarian tissue cryopreservation for fertility preservation: lessons learned from 545 cases. Hum Reprod.

[25] Jensen AK, Rechnitzer C, Macklon KT, Ifversen MR, Birkebaek N, Clausen N (2017). Cryopreservation of ovarian tissue for fertility preservation in a large cohort of young girls: focus on pubertal development. Hum Reprod.

[26] Modi DN, Sane S, Bhartiya D (2003). Accelerated germ cell apoptosis in sex chromosome aneuploid fetal human gonads. Mol Hum Reprod.

[27] Reynaud K, Cortvrindt R, Verlinde F, De Schepper J, Bourgain C, Smitz J (2004). Number of ovarian follicles in human fetuses with the 45, x karyotype. Fertil Steril.

[28] Magee AC, Nevin NC, Armstrong MJ, McGibbon D, Nevin J (1998). Ullrich-Turner syndrome: seven pregnancies in an apparent 45, X woman. Am J Med Genet.

[29] Hook EB, Warburton D (2014). Turner syndrome revisited: review of new data supports the hypothesis that all viable 45, X cases are cryptic mosaics with a rescue cell line, implying an origin by mitotic loss. Hum Genet.

[30] Hook EB (1977). Exclusion of chromosomal mosaicism: tables of 90%, 95% and 99% confidence limits and comments on use. Am J Hum Genet.

[31] Graff A, Donadille B, Morel H, Villy MC, Bourcigaux N, Vatier C (2020). Added value of buccal cell FISH analysis in the diagnosis and management of Turner syndrome. Hum Reprod.

[32] Gravholt CH, Backeljauw P (2017). New international Turner syndrome guideline: a multi-society feat. Eur J Endocrinol.

[33] Bouchlariotou S, Tsikouras P, Dimitraki M, Athanasiadis A, Papoulidis I, Maroulis G (2011). Turner's syndrome and pregnancy: has the 45, X/47, XXX mosaicism a different prognosis? Own clinical experience and literature review. J Matern Fetal Neonatal Med.

[34] Suzuki N (2015). Ovarian tissue cryopreservation using vitrification and/or in vitro activated technology. Hum Reprod.

[35] McLaughlin M, Albertini DF, Wallace WHB, Anderson RA, Telfer EE (2018). Metaphase II oocytes from human unilaminar follicles grown in a multi-step culture system. Mol Hum Reprod.

[36] Coccia ME, Rizzello F, Mariani G, Bulletti C, Palagiano A, Scarselli G (2011). Ovarian surgery for bilateral endometriomas influences age at menopause. Hum Reprod (Oxford, England).

[37] Bjelland EK, Wilkosz P, Tanbo TG, Eskild A (2014). Is unilateral oophorectomy associated with age at menopause? A population study (the HUNT2 Survey). Hum Reprod (Oxford, England).

[38] Hewitt JK, Jayasinghe Y, Amor DJ, Gillam LH, Warne GL, Grover S (2013). Fertility in Turner syndrome. Clin Endocrinol.

[39] van Hagen IM, Duijnhouwer AL, Ten Kate-Booij MJ, Dykgraaf RH, Duvekot JJ, Utens EM (2017). Wish to conceive and concerns to develop cardiovascular complications during pregnancy in patients with Turner syndrome. J Psychosom Obstet Gynaecol.

[40] Gravholt CH, Andersen NH, Conway GS, Dekkers OM, Geffner ME, Klein KO (2017). On behalf of the international Turner syndrome consensus group. Clinical practice guidelines for the care of girls and women with Turner syndrome: proceedings from the 2016 Cincinnati international Turner syndrome meeting. Eur J Endocrinol.

[41] Mazzanti L, Cacciari E, Bergamaschi R, Tassinari D, Magnani C, Perri A (1997). Pelvic ultrasonography in patients with Turner syndrome: age-related findings in different karyotypes. J Pediatr.

[42] Cleemann L, Holm K, Fallentin E, Skouby SO, Smedegaard H, Møller N (2011). Uterus and ovaries in girls and young women with Turner syndrome evaluated by ultrasound and magnetic resonance imaging. Clin Endocrinol (Oxf).

